# Adolescence as a turning point: for better and worse

**DOI:** 10.1007/s00787-012-0296-3

**Published:** 2012-06-22

**Authors:** Jan K. Buitelaar

**Affiliations:** 1 Department of Cognitive Neuroscience, Radboud University Nijmegen Medical Center, P.O. Box 9101, 6500 HB Nijmegen, The Netherlands; 2 Karakter Child and Adolescent Psychiatry, Nijmegen, The Netherlands

Adolescence is a transition period between childhood and adulthood that is characterised by major biological, psychological and social challenges and opportunities. The one-year prevalence of any psychiatric disorder in adolescents is considerable and ranges from 15 to 30 % [1]. Psychiatric disorders with onset in childhood or even earlier, such as autism spectrum disorders (ASD) and attention-deficit hyperactivity disorder (ADHD), show a strong persistence through adolescence until adulthood. However, the phenotypic expression of ASD and ADHD may change, and comorbid disorders as anxiety disorders, mood disorders and substance use may increasingly complicate their clinical presentation, diagnosis and management [2]. In addition, another substantial amount of psychopathology arises de novo during adolescence, among which the major disorders of adulthood as psychoses, bipolar illness, anxiety, mood, and personality disorders [3]. Further study of the key biological, psychological and social challenges of adolescence may therefore illuminate the understanding of risk and protective factors for both persistent childhood disorders as de novo psychopathology and inform strategies for early intervention and prevention.

One of more decades ago, biological explanations of adolescent turmoil were mainly phrased in terms of “raging hormones” [4]. The role of hormones has been established—no dispute about that—though the activating hormonal effects appear to be more modest than thought earlier. In the meantime, current interest has shifted to the study of how brain structure and function changes in adolescence, and how this may be related to onset and persistence of psychopathology. A central clinical characteristic of many adolescents is their impulsive and risk taking behavioural style. The biological mechanisms involved in this characteristic certainly are diverse and manifold. A cognitive neuroscience perspective describes risk taking behaviour as resulting from the dynamic interaction of the cognitive control system (medial/ventral prefrontal cortex), the reward system (nucleus accumbens), and the harm-avoidant system (amygdala) [5]. Increased risk taking might then be the consequence of either a weak control system, or an easily activated and pushing reward system, or a weak harm-avoidant system, or combinations of these. Let us present a snapshot of what is currently known about these systems in adolescence.

A longitudinal MRI study revealed that grey matter in the prefrontal cortex peaks in volume during early adolescence and then declines [6]. In contrast, white matter increases in volume through the adolescent years [7]. These and other findings indicate that the prefrontal cortex follows a protracted developmental time course. This may explain the slow maturation of functions that are mediated by the prefrontal cortex, such as inhibitory control, planning, and decision making [8]. In contrast to the rather slow and linear development of the prefrontal regions, data suggest that striatal brain regions underlying reward driven and impulsive behaviour may show a curvilinear developmental pattern with a peak inflection between 13 and 17 years [9]. This is of great interest, since the processing of reward-related stimuli primarily in the nucleus accumbens may relate to increased risk-taking behaviour in adolescents [5]. Note that, however, there exist two competing theories about the functional status of the reward system in adolescence. One hypothesis suggests that the nucleus accumbens is relatively hypo-responsive to rewards during adolescence, and an increase of reward-seeking behaviour is necessary to achieve the same activation as in adults. Another hypothesis says that, just the opposite, the striatal reward system is hyper-responsive, and this leads to greater reward-seeking behaviour [10]. The jury is still out, though the second hypothesis has more supporting evidence [10].

These differing developmental trajectories of frontal cortex and striatrum map onto the developmental differences seen in the onset of impulsive and reward seeking behaviours. Thus, impulsive and reward seeking behaviours have an early developmental onset, while these systems are still maturing, whereas the tension between late-developing prefrontal systems and more mature striatal systems during adolescence may lead to teenagers being more vulnerable to substances. Studies in healthy adolescents indeed show exaggerated striatal responsiveness with prefrontal activity intermediate between that of children and adults [9]. No studies have yet addressed this issue yet in developmental disorders as ADHD and ASD.

Let us point to the interesting results from experimental studies on contextual factors that impact cognitive control and decision making in adolescents and may be easily transferred to the clinical context. One is that adolescents are able to modify making risky choices when they are adopting the perspective of other persons. Thus, they tend to take less risky decisions for other persons than for themselves! Differences between self and other were larger in early than in late adolescence. At older age, adolescents seem to be able to better integrate others’ perspectives into one’s decision making [11]. Another finding is that high stress conditions were found to result in greater risky decision making compared to low stress conditions, but high versus low stress conditions did not affect response inhibition [12]. Also, do not forget that adolescents may sometimes be the victims rather than the aggressors, and suffer from distress, psychiatric problems and violent behaviours of their parents [13, 14].

New insights in the complexity of cognitive control functioning in adolescents were recently provided by an amazing paper of the IMAGEN cohort [15]. The well-known stop-signal reaction time task, which measures the ability to stop an ongoing response, was administered in a large sample of close to 1,900 14-year old adolescents while lying in the MRI scanner. The sample size was large enough to move beyond the typical regions of interest analysis and apply multivariate statistical approaches to the brain activation data. This allowed to identify not one, but seven distinct cortical and subcortical brain networks involved in successful inhibitions and not one but six distinct brain networks linked with failed inhibitions [15]. These brain networks were differentially associated with the presence of ADHD symptoms and with initiating drug use. Contrary to the expectations however, there appeared to be little overlap between the networks associated with ADHD symptoms and drug use, suggesting that these problems arise from different neurobiological pathways. Longitudinal follow-up studies are needed to disentangle the causal directions of these effects, i.e. whether drug use affects the activity in cognitive control networks, or vice versa, whether altered activity in control networks predisposes to drug use.

How does the harm-avoidance system factor in these equations between the prefrontal cortex and the striatum? Much less is known here. Studies of whether adolescents have similar, stronger or just weaker amygdala activations to fearful stimuli than adults have revealed conflicting findings [16]. Further developmental studies on the interactions, i.e. the strength of functional and structural connectivity between the prefrontal cortex, amygdala and striatum have to be conducted. This would permit to obtain a more full neuroscience perspective on the development of risky behaviours in adolescence and to evaluate the role of harm-avoidance system.

Not only biological but also complex psychological and social challenges occur in adolescence. These include becoming more independent from adults as parents and teachers, and being more subject to social evaluations by peers. Ferrin et al. [17] report in this issue of ECAP on the problems in treating adolescents with ADHD. Many of these adolescents tend not to adhere to the treatment recommended and stop taking medication. This may be due to various factors, such as fear for social stigma, worries about potential or perceived side effects of the medication, or struggling with the wish for autonomy and trying to manage “by yourself” with impulsivity, hyperactivity and inattention symptoms. Ferrin et al. decided to address this issue by developing and testing a new instrument that comes with two versions, one to be completed by adolescent patients themselves and one by their parents. The instrument explores patients’ and their families’ attitudes towards the treatments of ADHD. Identifying and quantifying their insights into the symptoms, their worries, fear for social stigma, and many more of these items will facilitate to raise these issues in the consultation room and have focused discussions about the pros and cons of the treatments recommended. This may ultimately lead to the development of more effective interventions for adolescents with ADHD. This is very much needed, given the results of the rather gloomy outcomes of patients with ADHD at the long-term follow-up of the MTA study [18] and other ADHD cohorts [19] indicating that adolescence is a turning point for better or worse.

Hawton et al. [20] describe the results of a multi-centre study on the descriptives and correlates of self-harm, defined here as intentional self-poisoning or self-injury. Rates of self-harm were found to increase rapidly from the age of 12 years onward, especially in girls. Correlates were somewhat different in boys and girls, with problems with alcohol, drugs and legal issues being more common in the boys, whereas difficulties in social relationships with families and friends being more common in girls. Self-harm is a complex symptom that may relate to deficient coping strategies, weak cognitive control functions, and intense affective and motivational problems that alone or in combination leads adolescents to decompensate. However, the issue needs to be placed in a broader context. The huge variation in the rates of self-injury between the different sites probably reflects additional effects of socio-economic disadvantage. Further, in spite of the existing of national guidelines in the United Kingdom for the assessment and management of patients with self-harm, rates of self-harm did not decline over the last period of time and <60 % of the patients went through a psychosocial assessment procedure.

The teenage brain is work in progress. We should continue in improving our understanding of what is going on the adolescents’ brains, minds, bodies and social world. We need to take the opportunity of the turning point of adolescence: change the worse into the better!
